# Tissue Clearing and Expansion Methods for Imaging Brain Pathology in Neurodegeneration: From Circuits to Synapses and Beyond

**DOI:** 10.3389/fnins.2020.00914

**Published:** 2020-10-05

**Authors:** Arnaldo Parra-Damas, Carlos A. Saura

**Affiliations:** ^1^Institut de Neurociències, Departament de Bioquímica i Biologia Molecular, Facultat de Medicina, Universitat Autònoma de Barcelona, Barcelona, Spain; ^2^Centro de Investigación Biomédica en Red Enfermedades Neurodegenerativas, Instituto de Salud Carlos III, Madrid, Spain

**Keywords:** tissue clearing, expansion microscopy, neuropathology, neurodegeneration, super-resolution microscopy

## Abstract

Studying the structural alterations occurring during diseases of the nervous system requires imaging heterogeneous cell populations at the circuit, cellular and subcellular levels. Recent advancements in brain tissue clearing and expansion methods allow unprecedented detailed imaging of the nervous system through its entire scale, from circuits to synapses, including neurovascular and brain lymphatics elements. Here, we review the state-of-the-art of brain tissue clearing and expansion methods, mentioning their main advantages and limitations, and suggest their parallel implementation for circuits-to-synapses brain imaging using conventional (diffraction-limited) light microscopy -such as confocal, two-photon and light-sheet microscopy- to interrogate the cellular and molecular basis of neurodegenerative diseases. We discuss recent studies in which clearing and expansion methods have been successfully applied to study neuropathological processes in mouse models and postmortem human brain tissue. Volumetric imaging of cleared intact mouse brains and large human brain samples has allowed unbiased assessment of neuropathological hallmarks. In contrast, nanoscale imaging of expanded cells and brain tissue has been used to study the effect of protein aggregates on specific subcellular structures. Therefore, these approaches can be readily applied to study a wide range of brain processes and pathological mechanisms with cellular and subcellular resolution in a time- and cost-efficient manner. We consider that a broader implementation of these technologies is necessary to reveal the full landscape of cellular and molecular mechanisms underlying neurodegenerative diseases.

## Introduction

Neurodegenerative diseases, including Alzheimer’s disease (AD), Parkinson’s disease (PD), Huntington’s disease (HD), amyotrophic lateral sclerosis (ALS), and frontotemporal dementia (FTD), among others, are incurable brain disorders that affect millions of people worldwide, causing enormous social and economic burdens. Despite global intensive research and development efforts centered on neurodegenerative diseases, there are currently no effective therapies that can revert or prevent their progression. A deeper understanding of the brain structure, function and their pathological alterations is necessary to identify and develop novel therapeutic strategies. This endeavor requires integrating evidence from multiple methodological approaches used in cellular and molecular neuroscience, including structural information at the subcellular and systems biology levels.

The fundamental relationship between the structure and function of biological systems implies that any dysfunction occurring under pathological conditions may be explained in terms of structural alterations. Although this may seem over-simplistic for a highly complex organ such as the brain (whose function is determined by the connectivity and activity patterns of diverse cell populations) indeed structural changes in specific cell types underlie almost every long-term brain function under a wide range of physiological and pathological conditions, including learning, memory, acquisition of motor skills, emotional and stress responses, neurodevelopmental and neuropsychiatric disorders -including addictions-, inflammation, aging, and neurodegeneration. In particular, altered synaptic structures are observed in many neuropsychiatric disorders, whereas early loss of synapses occurs in neurodegenerative dementias such as AD, long before neuronal death occurs (Penzes et al., 2011; Sheng et al., 2012). However, our overall knowledge about the structure-function relationship on the nervous system remains very limited (Lichtman and Denk, 2011). Thus, any progress made on basic and biomedical neuroscience research in this regard will surely impulse therapeutic developments for neurodegenerative brain disorders.

**FIGURE 1:**
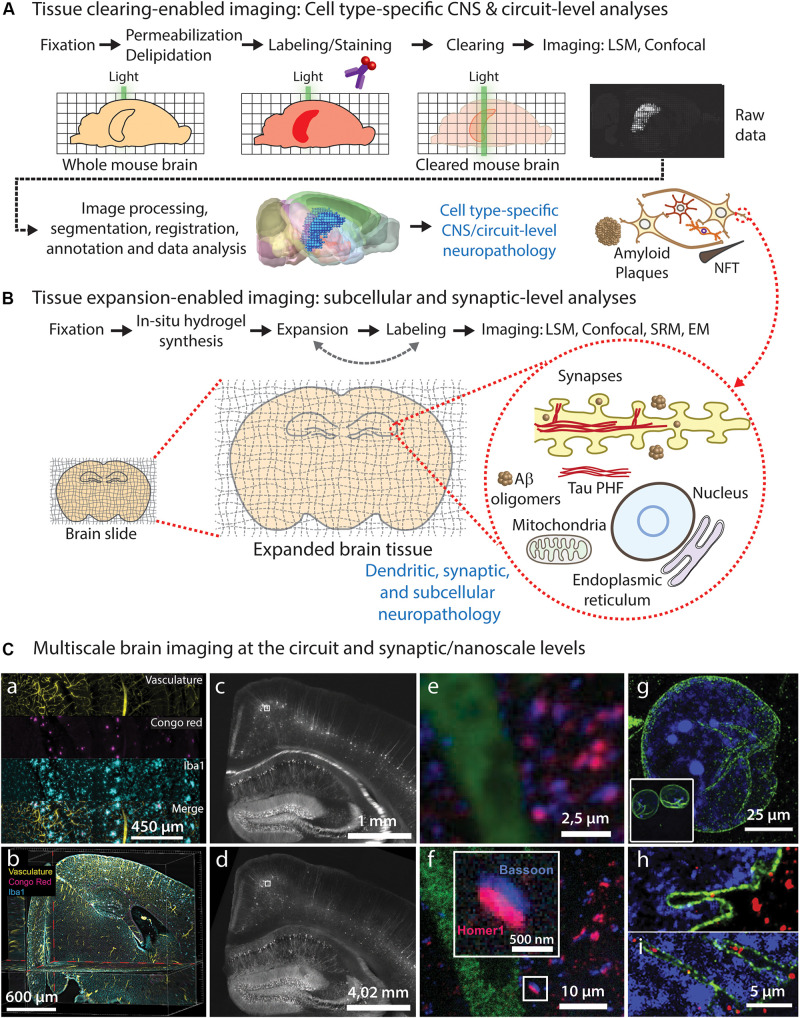
Brain tissue clearing and expansion approaches for neuropathological imaging. **(A)** Common steps required for whole-brain tissue clearing, imaging, and analysis of neuropathological features, allowing CNS-wide identification of vulnerable circuits and cell types. **(B)** Tissue expansion approaches can be used to study early pathological alterations caused by abnormal protein conformation, aggregation, and subsequent ultrastructural changes in specific subcellular compartments and organelles within affected cell types and circuits identified using tissue clearing. **(C)** Diffraction-limited light microscopy of cleared brain tissue allows imaging of large brain circuits at cellular resolution **(a–c)**, including analysis of neuropathological markers **(a,b)**, but it does not reach synaptic nanoscale resolution in non-expanded tissues **(e)**. On the contrary, expansion microscopy of tissues and cells **(d,f-i)** allows nanoscale imaging using diffraction-limited microscopes, enabling detailed visualization of synapses **(e)** and other subcellular structures such as nuclear envelope invaginations **(g–i)** in healthy **(h)** and ALS pathological conditions **(i)**. **(a,b)** LSFM images showing individual channels **(a)** and orthogonal optical planes **(b)** of a cleared transgenic AD mouse brain after staining of vasculature (yellow), Aβ plaques (Congo red; magenta) and microglia (Iba1; cyan) using the iDISCO protocol; adapted from Liebmann et al. (2016), with permission from Elsevier. **(c-f)** Epifluorescence images of a Thy1-YFP mouse brain stained with presynaptic (Bassoon, blue) and postsynaptic (Homer1, red) markers, before **(c,e)** and after **(d-f)** expansion microscopy; adapted from Chen et al. (2015), with permission from AAAS. **(g-i)** Confocal microscopy images of expanded human iPSC-derived motor neuron nuclei from healthy control **(h)** and ALS patient **(i)** [**(g)** inset: nuclear size before expansion], immunolabeled for LMNB1 (green) and DNA (blue); adapted from Ortega et al. (2020), with permission from Elsevier.

Understanding the cellular and molecular mechanisms underlying neurodegeneration requires unbiased analyses of the neuropathological features and structural alterations affecting heterogeneous cellular populations in specific (often multiple) brain circuits. Ideally, such imaging analyses must be performed throughout the entire volume of the intact brain, spinal cord and peripheral nerves. On the other hand, studying the impact of these pathological features at the individual cellular level also requires imaging small cellular and subcellular structures, such as synapses, organelles and protein complexes, which are often too small to be imaged using standard light microscopy. Therefore, an ideal comprehensive structural assessment should be performed throughout the entire spatial scale of the nervous system, from circuits to synapses. Here, we discuss recent advances on brain tissue clearing and expansion microscopy methods that can facilitate this endeavor in the context of neurodegeneration research, and propose a convenient imaging pipeline (Figure 1) that can be implemented using standard light microscopy, consisting on: (1) circuit-level assessment of neuropathological changes using tissue clearing (Figure 1A); and (2) nanoscale imaging of pathology-associated alterations in synaptic and subcellular compartments using expansion microscopy (Figure 1B).

## Tissue Clearing Methods for Circuit-Level Brain Imaging

Until recently, it was not possible to image deep brain structures using light microscopy without relying on histological sectioning, because the light transmitted through the sample is absorbed and scattered due to the different refractive indexes (RI) of cellular components, including lipids, proteins, and water. Consequently, the thickness of a brain section that can be efficiently imaged using confocal microscopy is limited to ∼150 μm, whereas two-photon microscopy can reach up to ∼1 mm from the sample surface, which is still not enough for imaging entire brain circuits. Tissue clearing methods homogenize the RI throughout the tissue, resulting in transparent samples that can be imaged from end to end using fluorescence light microscopy. Thus, cleared samples can be imaged using epifluorescence, confocal and two-photon microscopes or, more conveniently, by using light-sheet (selective plane illumination) fluorescence microscopy (LSFM) (Dodt et al., 2007; Hillman et al., 2019), which generates a thin laser sheet to illuminate a single plane in the entire sample, allowing high-speed and high-resolution imaging of large specimens, such as a whole mouse brain and even intact human organs (Zhao et al., 2020). While current light-sheet microscopes allow imaging of intact cleared brains from small animal models (such as mice, rats, pigs, and marmosets), imaging of the entire cleared human brain remains a major challenge.

A wide range of tissue clearing methods has been recently developed (Table 1), allowing simultaneous multicolor imaging of several targets in large sample volumes. Besides imaging fluorescent proteins, most of these methods allow labeling of multiple proteins and transcripts using fluorophore-conjugated antibodies, nanobodies, and RNA probes for fluorescent *in situ* hybridization (FISH) (Ueda et al., 2020). A detailed comparison of the fundamentals of tissue clearing methods is beyond the scope of this article since this has been the subject of several recent reviews (Richardson and Lichtman, 2015; Tainaka et al., 2016; Porter and Morton, 2020; Ueda et al., 2020). Nevertheless, before discussing the application of tissue clearing to neuropathological studies, we will first briefly mention the overall basis and the main advantages and limitations of the three major types of clearing methods: hydrophobic-, hydrophilic- and hydrogel-based clearing (Box 1 and Table 1).

**Advantages and limitations of the main tissue clearing technologies.**

**Table:** 

**Hydrophobic-based tissue clearing**	
Advantages	Very quick and reproducible protocols requiring simple immersion. Higher clearing performance (transparency).
Limitations	Loss of endogenous FP signal. Complete loss of lipids and associated biomolecules. May cause loss of proteins and nuclei acids. May require solvent-resistant materials and optics. Use of volatile and toxic solvents (require fume hoods and ventilated work areas).
**Hydrophilic tissue clearing**	
Advantages	Endogenous FP signal is preserved. Use of safer reagents. Compatible with standard materials and optics.
Limitations	May require longer incubation times. Protocols using high detergent concentrations may cause loss of lipids and associated biomolecules, proteins and nuclei acids.
**Hydrogel-based tissue clearing**	
Advantages	Endogenous FP signal is preserved. A higher proportion of biomolecules is retained. The brain anatomy and ultrastructure are better preserved.
Limitations	Longer and more complex protocols (compared with hydrophobic- and hydrophilic- methods) requiring *in situ* hydrogel polymerization. May require active (electrophoretic) labeling and clearing.

**TABLE 1:** Key features of the main tissue processing methods discussed.

Protocol	Fixation/Cross-linking	Permeabilization and Delipidation	Decolorization	Preservation of fluorescence from FP	Labeling/Staining	Clearing performance [RI], Reagent	Effect on tissue size	Max. # Resolution	Clearing time^†‡^¶	Application to neuropathology Related computational tools
**Hydrophobic (Organic Solvent) Tissue Clearing**										
BABB (Dodt et al., 2007)	Standard PFA perfusion	Strong: EtOH, DCM	-	Low (hours)	Thioflavin-S for Aβ plaques and lectin for vasculature (Huang et al., 2015).	Very high [1.56], BABB	Slight shrinkage	0.5-2 μm	7 days†	Visualization of Aβ plaques and vasculature in entire mouse brains (Huang et al., 2015).
3DISCO (Ertürk et al., 2012a, b)	Standard PFA perfusion	Strong: THF, DCM	-	Low (hours)	-	Highest [1.56], BABB	Shrinkage (∼30% volume)	0.5-2 μm	2 days†	Assessment of axonal degeneration and regeneration in spinal cord injury (Ertürk et al., 2012b).
iDISCO (Renier et al., 2014) iDISCO + (Renier et al., 2016) https://idisco.info/	Standard PFA perfusion	Strong: Methanol, DCM	H_2_O_2_	Low (hours)	Antibodies, Congo red for Aβ plaques	Highest [1.56], DBE	Minimal	0.5-2 μm	7-14 days†	Evaluation of Aβ pathology in AD transgenic mice and human brains; automated quantification of Aβ plaques with ClearMAP (Liebmann et al., 2016). Imaging of temporal/spatial progression of tau neuropathology in EC-tau mice (Fu et al., 2016).
u/vDISCO (Pan et al., 2016) (Cai et al., 2019) discotechnologies.org	Standard PFA perfusion	Strong: *tert*-Butanol, THF DCM/Triton-X100	Aminoalcohol	Acceptable (hours-days)	Nanobodies (vDISCO; 2-3 d. active or 5-7 d. passive staining)	Highest [1.56], BABB-D	Shrinkage (∼35–55% volume)	1-2 μm	5 days†	Machine learning pipeline for identification and quantification of nanobody-labeled cells (Pan et al., 2019).
SHANEL* (Zhao et al., 2020) discotechnologies.org	PFA perfusion of entire human organs	Strong: CHAPS, DCM	CHAPS/NMDEA	N/A	Antibodies, Methoxy-X04 for labeling Aβ plaques	Very high [1.56], BABB	Shrinkage (∼30–45% volume)	1-2 μm	1- > 3^‡^weeks	Detection of amyloid plaques in human brain tissue (POC); deep learning-based pipeline for 3D reconstruction and data analysis (Zhao et al., 2020).
**Hydrophilic Tissue Clearing**										
CUBIC 1/2 (Susaki et al., 2014) cubic.riken.jp	Standard PFA perfusion	Strong: Sca*l*eCUBIC-1 (15% Triton-X100 + 25% Aminoalcohol + 25% Urea)	Aminoalcohol	High	Antibodies	High [1.49], ScaleCUBIC-2 (urea + aminoalcohol + sucrose)	Slight expansion	0.5-2 μm	7-14 days†	Visualization of detailed neuronal morphology and Aβ plaques using Golgi and Thioflavin-S staining in AD mice (POC) (Vints et al., 2019).
Updated CUBIC protocols (Tainaka et al., 2018) cubic.riken.jp	Standard PFA perfusion	Strong: CUBIC-L (10% Triton-X100 + 10% Aminoalcohol)	Aminoalcohol	High	Antibodies	High [1.52], CUBIC-R/RA (antipyrine, nicotinamide)	Slight expansion	0.5–2 μm	5–11 days†	–
Sca*l*eS* (Hama et al., 2015).	Standard PFA perfusion	Mild: urea + sorbitol + DMSO	–	High	Antibodies, Lectin	High [1.44] (urea + sorbitol + DMSO)	Minimal	0.5–2 μm	2–5 days for mm-thick slides.	Evaluation of Aβ pathology, microglia and EM ultrastructure in AD transgenic mice and human AD brains (Hama et al., 2015).
**Hydrogel-Based Tissue Clearing**										
CLARITY (Chung et al., 2013; Tomer et al., 2014) clarityresourcecenter. com	PFA/acrylamide/bis-acrylamide	Strong: 4% SDS	–	Temperature-dependent: preserved at 37°C	Antibodies	Good [1.45], FocusClear	Minimal	0.5–2 μm	1 (active)-3 (passive) weeks†	Visualization of Aβ plaques and tangles in human AD brain (POC) (Ando et al., 2014). Identification of fragmented nigrostriatal axons in PD mice (Nordström et al., 2015). Visualization of Lewy pathology in human brain (POC) (Liu et al., 2016).
SWITCH* (Murray et al., 2015) chunglabresources.com	PFA, Glutaraldehyde	200 mM SDS	–	Temperature-dependent: preserved at 37°C requiring longer clearing	Antibodies, Lectin	High, [1.47], diatrizoic acid, *n*-methyl-D-glucamine, iodixanol	Minimal	0.5–2 μm	2–4 weeks†	Assessment of Aβ deposits in 5xFAD mice, revealing early Aβ accumulation in subcortical areas (Gail Canter et al., 2019).
SHIELD* (Park et al., 2019) chunglabresources.com	PFA, Polyepoxide	300 mM SDS	-	Time/temperature-dependent: preserved at 45°C	Antibodies, FISH, Lectin	High, [1.46], Iodixanol- or Iohexol-based Protos media	Minimal	0.5–2 μm	1 (active) -4 (passive) weeks†	Quantitative evaluation of Aβ pathology in 5xFAD mice exposed to gamma sensory stimulation (Martorell et al., 2019).
**Tissue Expansion**										
ExM/ProExM/iExM (Chen et al., 2015; Chang et al., 2017; Asano et al., 2018) expansionmicros copy.org	PFA, acrylamide/bis-acrylamide/sodium acrylate Acryloyl-X	5% Triton X-100, 1% SDS (for post expansion staining)	–	Partial, only digestion-resistant FP	Antibodies, FISH	High, [∼1.33], (water)	20 × (iExM) 4.5 × (ExM) Expansion (3.5 × for FISH)	∼25 nm (iExM) or 60 nm (ExM)	1-2 days ¶	Identification of perivascular c43 accumulation on astrocytic endfeet in human brain epilepsy (Deshpande et al., 2017). Identification of eRF1 accumulation within nuclear envelope invaginations in iPSC-derived neurons from ALS patients (Ortega et al., 2020).
ExPath* (Zhao et al., 2017; Bucur et al., 2020)	PFA, formalin or acetone. Acrylamide/bis-acrylamide/sodium acrylate	0.1% Triton X-100	–	N/A	Antibodies, DNA-FISH	High, [∼1.33], (water)	4.5×	∼60 nm	4 h (≤5 μm) – 2 days ¶	–
MAP (Ku et al., 2016) chunglabresources.com	PFA, acrylamide/bis-acrylamide/sodium acrylate	200 mM SDS	–	Fluorescence is lost but epitopes are preserved	Antibodies	[1.47] pre-expansion. [∼1.33] (water) after expansion.	Fourfold linear expansion	∼60-nm	∼1 week ¶	–
X10 ExpM (Truckenbrodt et al., 2018, 2019)	Sodium acrylate, *N,N*-dimethyl acrylamide, Acryloyl-X	0.1% Triton X-100	–	Fluorescence is lost but epitopes are preserved	Antibodies	High, [∼1.33], (water)	Tenfold expansion	∼25 nm	3 days ¶	–
SHIELD-MAP (3x linear expansion) (Park et al., 2019) chunglabresources.com	PFA, Polyepoxide acrylamide/bis-acrylamide/sodium acrylate/VA-044	300 mM SDS	–	Time/temperature-dependent. 27-fold signal reduction after expansion.	Antibodies	DI water for expansion.	Threefold linear expansion	?	∼2 weeks ¶ (1–3 mm Slices)	–

^#^Lateral resolution using standard (diffraction-limited) light microscopy. *Optimized for human tissue. ^†^For an intact mouse brain. ^‡^For ∼1 cm^3^ human brain tissue. ¶Expansion of brain slices. DCM, dichloromethane. CHAPS, 3-[(3-cholamidopropyl)dimethylammonio]-1-propanesulfonate. BABB, benzyl alcohol + benzyl benzoate (1:2). BABB-D, BABB + Dibenzyl ether. SDS, sodium dodecyl sulfate. POC, proof-of-concept application.

### Hydrophobic-Based Tissue Clearing

Hydrophobic (organic solvent-based) clearing methods rely on tissue dehydration, delipidation and permeabilization using organic solvents such as ethanol (Dodt et al., 2007), methanol (Renier et al., 2014), tetrahydrofuran (THF) (Ertürk et al., 2012a), and tert-butanol (Pan et al., 2016). Most protocols involve simple tissue immersion (passive clearing) in a graded series of increasing solvent concentration. However, active clearing protocols in which the solvents are perfused through the circulatory system allow clearing entire rodent bodies (Pan et al., 2016) and large organs such as the human brain (Zhao et al., 2020). Further incubation in dichloromethane (DCM) may improve tissue delipidation in large samples, at the expense of decreasing fluorescence from fluorescent proteins (FP) (Ertürk et al., 2012a). After dehydration, samples are immersed in a clearing solution consisting of a mixture of benzyl alcohol and benzyl benzoate (BABB) (Dodt et al., 2007) or dibenzyl ether (DBE) (Ertürk et al., 2012a), to achieve RI homogenization and thus transparency.

### Hydrophilic Tissue Clearing

Tissue clearing may also be achieved using hydrophilic reagents, including different detergents (Triton-X100, saponin, sodium dodecyl sulfate -SDS-, etc.) for permeabilization and delipidation, and high-refractive index aqueous solutions containing sugars (fructose, sucrose, sorbitol) (Ke et al., 2013), urea (Hama et al., 2011), aminoalcohols (Susaki et al., 2014), or different combinations of the former (Table 1).

### Hydrogel-Based Tissue Clearing

Another main approach to clear tissues consists of embedding the samples on a hydrogel matrix to create a cross-linked tissue/gel hybrid containing the fixed proteins and RNA (Gradinaru et al., 2018). Ionic detergents are used for delipidation, which can be enhanced by electrophoresis without causing significant loss of biomolecules.

## Tissue Clearing Applied to Neuropathology and Neurodegeneration

Despite affecting specific brain circuits, neurodegenerative diseases share several common pathophysiological features, including accumulation of protein aggregates forming characteristic neuropathological lesions. AD neuropathology is characterized by accumulation of extracellular amyloid plaques and intraneuronal neurofibrillary tangles (NFT), formed by aggregated amyloid-β (Aβ) peptides and hyperphosphorylated tau protein, respectively. α-synuclein inclusions, known as Lewy bodies, are found in PD and Lewy body dementias, while mutant huntingtin (mHTT) aggregates are formed in HD, and TAR DNA-binding protein 43 (TDP-43) aggregates are found in ALS and FTD brains (Nguyen et al., 2018). Some of these lesions (like aggregated tau, Lewy bodies and TDP-43 aggregates) are found in more than one disease, suggesting common underlying molecular mechanisms. Furthermore, most of these neuropathologies propagate following a specific anatomical pattern through connected brain circuits (Brettschneider et al., 2015), stressing the necessity of evaluating neuropathology at large spatial scales across multiple brain regions.

Recent studies have applied tissue clearing methods to evaluate some of these neuropathological hallmarks in large human brain samples and entire brains from transgenic mouse models. Huang and colleagues used the fluorescent dye Thioflavin-S for staining Aβ plaques, combined with vasculature labeling using fluorophore-conjugated lectin and BABB clearing of entire mouse brains (Dodt et al., 2007), to show that deletion of the orphan receptor GPR3 reduces the number and volume of amyloid plaques in an AD knock-in mouse model (*App*^*NL–F/NL–F*^) (Huang et al., 2015). Hama et al. (2015) applied their Sca*l*eS tissue clearing method to visualize Aβ plaques, microglia and brain ultrastructure in entire mouse brain hemispheres and in 1–2 mm-thick sections from *App*^*NL–F/NL–F*^ mice and human AD brains, using dye- (PP-BTA-1) and antibody- (6E10) labeling of plaques, neurons (NeuN) and microgia (Iba1). Compared to other clearing methods such as CUBIC, 3DISCO, and PACT, Sca*l*eS preserved the brain ultrastructure, allowing detection of postsynaptic densities and membranes using electron microscopy. A recent study used a modified Golgi staining combined with Thioflavin-S and CUBIC-based clearing to image detailed neuronal morphology along with Aβ plaques in brain sections and blocks from *App*^*NL–F/NL–F*^ mice (Vints et al., 2019).

The hydrogel-based CLARITY method has also been used to visualize amyloid plaques and NFT in human AD brain samples (Ando et al., 2014), fragmented nigrostriatal axons in a PD mouse model (Nordström et al., 2015), and Lewy pathology in a human PD brain (Liu et al., 2016), relying on antibody labeling for detecting neuropathology and specific cell types, and confocal microscopy for imaging. Imaging of amyloid plaques revealed sparse accumulation and diversity of 3D structures, including dense/focal and diffuse deposits, whereas tau accumulated in discontinuous neuritic processes associated with dense/mature plaques (Ando et al., 2014) which were previously reported using conventional histology and confocal microscopy (Knowles et al., 1999). The immunolabeling and solvent-based clearing method iDISCO has also been used to evaluate tau pathology and Aβ plaques using antibodies and Congo red labeling in APP_swe_/PSEN1ΔE9 and 3xTg-AD transgenic mice and human AD brains (Liebmann et al., 2016). The authors also developed a computational pipeline based on automated detection of cells and plaques from light-sheet images and further mapping of these features to the Allen Brain Atlas, enabling fast quantification of plaques in different brain regions (Liebmann et al., 2016). iDISCO+ (Renier et al., 2016), an improved version of iDISCO, was successfully applied for whole-brain immunolabeling and 3D imaging of the temporal and spatial progression of tau neuropathology in entorhinal cortex (EC)-tau transgenic mice, revealing age-dependent tau pathology not only in entorhinal and hippocampal regions but also in neocortical areas, which was not previously detected by classical 2D histology in this model (Fu et al., 2016).

More recently, SHIELD-based clearing (Park et al., 2019) of entire mouse brains was used to show that auditory plus visual gamma sensory stimulation reduces the number and volume of amyloid plaques (detected by Aβ immunolabeling) in 6-month-old 5xFAD mice, revealing a greater effect of sensory stimulation on pathology across broad cortical regions than observed with conventional histology (Martorell et al., 2019). Similarly, SWITCH whole-brain immunolabeling and clearing (Murray et al., 2015) were used to monitor the progression of Aβ deposits in 5xFAD mice, revealing early Aβ accumulation in subcortical areas and area-specific aggregation correlating with electrophysiological changes (Gail Canter et al., 2019). Finally, a novel permeabilization and clearing approach, named SHANEL, allowed labeling of cm-thick human brain tissue using conventional antibodies, and was used to detect amyloid plaques in human brains labeled with the Congo red derivative methoxy-X04 (Zhao et al., 2020).

Taken together, these studies have demonstrated the feasibility of using tissue clearing approaches for achieving volumetric imaging and quantitative assessment of neuropathological hallmarks in AD and PD.

## Nanoscale Imaging Using Super-Resolution Expansion Microscopy

Studying neuropathology-associated alterations in the molecular composition and ultrastructure of the brain is essential for understanding pathogenic mechanisms underlying neurodegenerative diseases. While electron microscopy achieves the highest spatial resolution for ultrastructural analyses, it still lacks the molecular specificity and throughput provided by multicolor fluorescence microscopy, which is required for simultaneous imaging of multiple targets, including visualizing cell-type-specific nanostructures and/or signaling molecules within a pathological context. However, the minimum size of the structures that can be resolved using conventional fluorescence light microscopy is constrained by the light diffraction limit (about 250 nm, depending on the specific excitation wavelength), which precludes detailed imaging of specific protein complexes, individual synapses and other subcellular structures. Several optical super-resolution microscopy (SRM) methods have been developed to overcome diffraction limitation, including stimulated emission depletion (STED) microscopy, photoactivation localization microscopy (PALM), structured illumination microscopy (SIM), and stochastic optical reconstruction microscopy (STORM), among others (Tønnesen and Nägerl, 2013; Schermelleh et al., 2019). Depending on the specific approach and imaging setup, optical SRM can resolve sub-diffraction size structures ranging from 30 to 100 nm (lateral resolution), enabling detailed visualization of subcellular structures, individual synapses and protein complexes. Interestingly, recent studies have combined the advantage of reduced light scattering (transparency) provided by tissue clearing methods, with SRM to achieve sub-diffraction volumetric brain imaging, revealing novel synaptic structural and functional insights. For instance, Ke et al. (2016) developed a tissue clearing solution (SeeDB2) which, combined with STED SRM, allowed synaptic-resolution imaging through thick brain samples (up to 120 μm). More recently, Hruska et al. (2018) combined CUBIC tissue clearing and STED SRM to demonstrate the occurrence of discrete synaptic nanomodules (∼80 nm in size) in the mouse somatosensory cortex.

However, SR microscopes are not widely available to most biomedical research labs, are expensive, and may require expert personnel to operate. Moreover, multicolor imaging may be challenging for some setups. A clever solution to overcome the light diffraction limit is to increase the size of the specimens to image (Chen et al., 2015). Three main independent approaches have been developed to increase the size of biological tissues: expansion microscopy (ExM) (Chen et al., 2015); magnified analysis of the proteome (MAP) (Ku et al., 2016); and X10 (Truckenbrodt et al., 2018) (Table 1). These methods use swellable hydrogels that are cross-linked throughout the tissue to induce isotropic expansion of the samples, allowing super-resolution imaging of the expanded specimens using conventional light microscopes [Figures 1B,C(c–i)]. Current tissue expansion methods allow isotropic expansion of tissues up to ∼4.5x (Ku et al., 2016; Tillberg et al., 2016) or 10x (Truckenbrodt et al., 2018) for single-step expansion protocols and up to ∼20x for iterative expansion protocols (Chang et al., 2017), enabling detailed nanoscale imaging of subcellular structures with up to ∼25-nm-resolution using conventional confocal microscopy (Table 1). These methods are easy to implement and optimize (compared with optical SRM), require commercially available reagents, and are compatible with multicolor immunostaining and FISH (Tillberg et al., 2016; Asano et al., 2018).

Moreover, expanded samples can be conveniently imaged using confocal microscopes -which are widely available- and provide enough three-dimensional resolution to enable cost-efficient super-resolution imaging of the brain tissue (Tillberg and Chen, 2019; Wassie et al., 2019). Each tissue expansion approach has its advantages and limitations related to preserving endogenous fluorescence, epitopes, and reduction of fluorescence signal after expansion (Table 1). In this regard, performing tissue immunolabeling after expansion may yield higher fluorescence signals, although fewer epitopes can be detected with this strategy (Asano et al., 2018).

## Insights From SRM in Neurodegeneration Research

Besides the evident relevance of assessing synaptic structural and molecular alterations in brain diseases, nanoscale imaging can also provide crucial information on other altered cellular processes. For instance, STORM and STED SRM have been applied to study the aggregation states of Huntingtin exon 1, including the formation of inclusion bodies and other fibrillary species in living and fixed cells (Duim et al., 2014; Sahl et al., 2016), while SIM SRM has been used to study the interaction of mHTT aggregates with transcription factors (Li et al., 2016), which could mediate altered gene expression in HD (Giralt et al., 2012). Different optical SRM approaches have helped to image both Aβ and tau aggregates *in vitro* and in cellular models (Pinotsi et al., 2016; Schierle et al., 2016), while STED SRM was used to image immunolabelled tau filaments in postmortem AD brain sections (50 μm) at ∼80 nm resolution (Benda et al., 2016). A recent study used STED SRM to identify disruption of the lamin nucleoskeleton in a *Drosophila* model of tau pathology and in human postmortem AD brain tissue, suggesting that lamin dysfunction contributes to tau-mediated neurodegeneration (Frost et al., 2016). STORM and STED SRM have also been used to visualize the presenilin-1/γ-secretase complex, which is responsible for the proteolytic cleavage of the amyloid precursor protein (APP) that releases Aβ, in both the pre- and post-synaptic compartments of cultured mouse neurons (Schedin-Weiss et al., 2016). More recently, SIM and PALM were used to study the subunit composition and activity of the γ-secretase complex at the plasma membrane in living cells, revealing complex dynamics involving association with specific substrates (Escamilla-Ayala et al., 2020).

Early after its development, protein-retention ExM (ProExM) (Tillberg et al., 2016) was used to identify “striosome–dendron bouquets” structures formed by intertwined striosomal axons and dopamine-containing dendrites as part of the dopamine-containing nigral system, which may be relevant for motor and neurodegenerative disorders (Crittenden et al., 2016). Deshpande et al. (2017) applied ProExM to human CA1 hippocampal tissue from temporal lobe epilepsy patients to study the subcellular localization of perivascular Connexin-43 (Cx43; GJA1), revealing specific Cx43 accumulation on the parenchymal side of astrocytic endfeet, compared to endothelial or pericytic accumulation. ProExM has also been applied to HeLa cells to study the spatial organization of autophagy-related HSPB1 protein and SQSTM1/p62 bodies in the context of peripheral neuropathy (Haidar et al., 2019). More recently, ProExM was used to show that the protein eRF1 accumulates within the cytosolic side of nuclear envelope invaginations identified in induced pluripotent stem cell (iPSC) neurons derived from ALS patients (Ortega et al., 2020) [Figures 1C(g–i)].

Taken together, these studies demonstrate that -similar to optical SRM- expansion-SRM has the potential to deliver relevant structural insights about molecular and cellular mechanisms mediating neuropathology and neurodegeneration. Broader implementation of these methods may be of great value for studying interactions between pathological protein conformations and organelles/membranes, which could mediate aggregation and downstream neurodegenerative processes (Shrivastava et al., 2017).

## Potential Applications of Clearing and Expansion Methods in Neurodegeneration Research

Based on the above considerations, we propose a general straightforward pipeline for structural and molecular analysis of neuropathological and neurodegenerative brain tissues, from circuits to synapses, relying on the capabilities offered by tissue clearing and expansion methods (Figure 1). This consists of: (1) Tissue clearing-enabled unbiased imaging of whole brains or specific brain regions; and (2) SR-expansion nanoscale imaging of subcellular compartments from specific cell-types within affected circuits. The first systems/circuit-level approach has several remarkable advantages compared with standard sectioning histology: first, it preserves the full anatomical information, some of which is lost during tissue sectioning. Second, it allows unbiased and automated imaging and data analysis of the whole brain, reducing experimental errors and favoring reproducibility. Third, the time required for tissue processing, labeling, imaging, and data analysis can be significantly reduced, especially when working with large experimental groups if automated imaging and data analysis pipelines are implemented (Table 1).

Despite these clear advantages, up to date, most tissue clearing studies have provided limited biological information or only proof-of-concept data on neuropathology and neurodegeneration, particularly in human brain samples (Table 1). However, we think that neuropathological studies can leverage tissue clearing methods to deliver insightful systems biology-level information on structural and functional alterations. For instance, tissue clearing approaches may be used to identify relevant brain circuits showing pathology-induced alterations in the activity and connectivity of specific cell-types, by performing simultaneous analysis of neuropathological hallmarks, cell type-specific and synaptic markers, as well as indicators of neuronal/astrocytic excitability (i.e., immediate early genes such as *c-fos* and *Arc*) or glial reactivity (GFAP, CD45). Indeed, tissue clearing methods have been successfully applied to study brain-wide neuronal activity (Renier et al., 2016; Sylwestrak et al., 2016; Murakami et al., 2018; Park et al., 2019) and -separately- AD neuropathology in mouse models (Table 1). These approaches could be combined to study how different neuropathologies affect the activity of specific cell populations in the whole brain, which could shed light into the cellular mechanisms mediating selective/differential vulnerability of different circuits and neuronal/glial subtypes during neurodegenerative diseases (Fu et al., 2018; Forrest et al., 2019). In particular, automated mapping of brain activity based on detection of neuronal activity-induced immediate early genes (Renier et al., 2016) can be combined with assessment of neuropathological features in neurodegeneration or AD mouse models (Liebmann et al., 2016) to obtain physiological functional readouts of circuit- and cell-specific vulnerability to AD neuropathology, and to evaluate potential therapeutic strategies. Different computational methods have been developed to achieve automated identification and quantification of cells and neuropathological hallmarks, which can be of high value in such studies (Table 1).

The former circuit-level imaging approaches need to be complemented with SR-expansion nanoscale imaging to further characterize the synaptic and subcellular ultrastructural alterations occurring under neuropathological conditions, and to investigate potential molecular and cellular mechanisms. Thus, cell-type-specific molecular mechanisms mediating alterations in synaptic and subcellular structures (such as altered mRNA and protein localization, local synaptic translation, protein-protein interactions, and pathological protein aggregation, among others) could be approached using expansion SRM (Figure 1B). Since SRM methods (including expansion microscopy) have already revealed key molecular mechanisms in physiological and neurodegenerative contexts, it is reasonable to pursue a broader implementation of tissue expansion technologies for studying cellular and molecular mechanisms in neurodegeneration research.

## Concluding Remarks

The astonishing development of tissue clearing and expansion technologies in the last few years suggests that there is still plenty of room for improvement. Future advances in tissue chemistry and optics (Chakraborty et al., 2019; Gwosch et al., 2020) together with recent initiatives to guide the building of open-source light-sheet microscopes (Voigt et al., 2019) will likely facilitate their applicability, while increasing the current resolution and the range of biomolecules (i.e., lipids, sugars), modifications (acetylations, methylations, sumoylation, etc.) and processes (interactions, synthesis, degradation, and aggregation) that can be imaged in fixed samples. On the other hand, recent advances in volumetric imaging of bio-microelectromechanical systems (bioMEMS microdevices) using LSFM opens a promising avenue for achieving 3D and sucellular imaging of *in vitro* model systems under highly controlled experimental conditions (Albert-Smet et al., 2019). Computational developments, including neuropathology-optimized data analysis tools and implementation of standardized pipelines, data formats, and data-sharing platforms can further aid quantitative assessments and systems-biology level interpretation of the vast amount of brain/circuit-wide imaging data that can be generated, as well as integration with functional and cell-specific omics data (Vandenberghe et al., 2018; Hériché et al., 2019). Moreover, incorporation of other structural and functional imaging technologies [such as positron emission tomography (PET), functional magnetic resonance imaging (fMRI), multi-photon microscopy, optical super-resolution imaging, cryo-EM and cryo-electron tomography, among others] is needed to confirm and complement the findings obtained by tissue clearing and expansion methods, as well as to achieve a complete imaging assessment throughout the entire spatial scale of the brain, which remains one of the most challenging tasks of contemporary neuroscience (Lichtman and Denk, 2011; Koning et al., 2018). However, we think that tissue clearing and expansion technologies have already reached enough maturity to be generally embraced by the neurodegeneration research community, and as such, they can be readily implemented to address the underlying biology and pathogenic mechanisms of neurodegeneration, as demonstrated by relevant recent studies (Gail Canter et al., 2019; Martorell et al., 2019). This certainly will enable biological discoveries of great translational impact in the near future.

## Author Contributions

AP-D conceived the study and designed the figures. AP-D and CS wrote the manuscript. Both authors contributed to the article and approved the submitted version.

## Conflict of Interest

The authors declare that the research was conducted in the absence of any commercial or financial relationships that could be construed as a potential conflict of interest.
